# Oncologic Outcomes of Salvage Abdominoperineal Resection for Anal Squamous Cell Carcinoma Initially Managed with Chemoradiation

**DOI:** 10.3390/jcm13082156

**Published:** 2024-04-09

**Authors:** Roni Rosen, Felipe F. Quezada-Diaz, Mithat Gönen, Georgios Karagkounis, Maria Widmar, Iris H. Wei, J. Joshua Smith, Garrett M. Nash, Martin R. Weiser, Philip B. Paty, Andrea Cercek, Paul B. Romesser, Francisco Sanchez-Vega, Mohammad Adileh, Diana Roth O’Brien, Carla Hajj, Vonetta M. Williams, Marina Shcherba, Ping Gu, Christopher Crane, Leonard B. Saltz, Julio Garcia Aguilar, Emmanouil Pappou

**Affiliations:** 1 Department of Surgery, Memorial Sloan Kettering Cancer Center, New York, NY 10065, USAffquezad@gmail.com (F.F.Q.-D.); smithj5@mskcc.org (J.J.S.);; 2 Department of Epidemiology and Biostatistics, Memorial Sloan Kettering Cancer Center, New York, NY 10065, USA; 3 Department of Medicine, Memorial Sloan Kettering Cancer Center, New York, NY 10065, USAgup@mskcc.org (P.G.);; 4 Department of Radiation Oncology, Memorial Sloan Kettering Cancer Center, New York, NY 10065, USA; romessep@mskcc.org (P.B.R.);; 5 Department of Computational Oncology, Memorial Sloan Kettering Cancer Center, New York, NY 10065, USA

**Keywords:** anal cancer, salvage APR, combined modality treatment

## Abstract

**Background:** Abdominoperineal resection (APR) has been advocated for persistent or recurrent disease after failure of chemoradiation (CRT) for anal squamous cell cancer (SCC). Treatment with salvage APR can potentially achieve a cure. This study aimed to analyze oncological outcomes for salvage APR in a recent time period at a comprehensive cancer center. **Methods:** A retrospective review of all patients who underwent APR for biopsy-proven persistent or recurrent anal SCC between 1 January 2007 and 31 December 2020 was performed. Patients with stage IV disease at the time of initial diagnosis and patients with missing data were excluded. Univariate analysis was used with a chi-square test for categorical variables, and non-parametric tests were used for continuous variables. Kaplan–Meier survival analysis was performed to evaluate disease-specific (DSS), post-APR local recurrence-free (RFS), and disease-free survival (DFS). **Results:** A total of 96 patients were included in the analysis: 39 (41%) with persistent disease and 57 (59%) with recurrent SCC after chemoradiation had been completed. The median follow-up was 22 months (IQR 11–47). Forty-nine patients (51%) underwent extended APR and/or pelvic exenteration. Eight (8%) patients developed local recurrence, 30 (31%) developed local and distant recurrences, and 16 (17%) developed distant recurrences alone. The 3-year DSS, post-APR local recurrence-free survival, and disease-free survival were 53.8% (95% CI 43.5–66.5%), 54.5% (95% CI 44.4–66.8%), and 26.8% (95% CI 18.6–38.7%), respectively. In multivariate logistic regression analysis, positive microscopic margin (OR 10.0, 95% CI 2.16–46.12, *p* = 0.003), positive nodes in the surgical specimen (OR 9.19, 95% CI 1.99–42.52, *p* = 0.005), and lymphovascular invasion (OR 2.61 95% CI 1.05–6.51, *p* = 0.04) were associated with recurrence of disease. Gender, indication for APR (recurrent vs. persistent disease), HIV status, extent of surgery, or type of reconstruction did not influence survival outcomes. Twenty patients had targeted tumor-sequencing data available. Nine patients had PIK3CA mutations, seven of whom experienced a recurrence. **Conclusions:** Salvage APR for anal SCC after failed CRT was associated with poor disease-specific survival and low recurrence-free survival. Anal SCC patients undergoing salvage APR should be counseled that microscopic positive margins, positive lymph nodes, or the presence of lymphovascular invasion in the APR specimen are prognosticators for disease relapse. Our results accentuate the necessity for additional treatment strategies for the ongoing treatment challenge of persistent or recurrent anal SCC after failed CRT.

## 1. Introduction

Anal cancers are rare, comprising about 0.5% of all new cancer diagnoses in the United States in 2023, with squamous cell carcinoma being the most common subtype [1]. Prior to the 1980s, patients with anal SCC would routinely undergo abdominoperineal resections (APR), with considerable morbidity. Since the Nigro protocol was first published in 1974, multiple trials have shown improved disease-free survival and colostomy-free survival with combined modality therapy, making chemoradiation with mitomycin-c and 5-FU the standard of care for locoregional disease today [2,3,4,5,6]. Combined modality therapy offers a long-term survival of up to 90% for patients without distant metastases [7,8]. As the incidence of anal squamous cell carcinoma increased over time, the treatment of local recurrence and persistent disease after combined modality therapy has become a treatment challenge [9].

After multimodal therapy, local failure—defined as persistent disease after 6 months, progression of disease, or recurrent disease—has been described in up to 30% of patients [2,5,10,11]. Currently, the mainstay of treatment for locoregional failure after chemoradiation for anal SCC is APR, with little proven benefit of adjuvant chemotherapy or radiation. Surgical outcomes feature high morbidity rates of up to 80%, with patients commonly experiencing complications like perineal dehiscence or infection [12]. Due to the rarity of the disease, reports of the oncologic outcomes of salvage APR are sparse and heterogenous due to small sample sizes and varying CRT regimens [11,13,14,15].

The aim of the present report is to analyze oncological outcomes for salvage APR for persistent/recurrent anal SCC in a contemporary timeframe at a comprehensive cancer center and identify potential predictors of poor prognosis.

## 2. Methods

### 2.1. Patient Selection

This retrospective review was approved by the institutional review board of Memorial Sloan Kettering Cancer Center (MSK) with a waiver of informed consent. A retrospective review identified patients who underwent APR for biopsy-proven anal SCC at MSK from January 2007 to December 2020. Patients with stage IV disease, synchronous cancer, or lack of follow-up were excluded. Demographic and treatment data were obtained via chart review, including age, surgery type, indication for surgery, HIV status, treatment regimen, pathology results, and oncologic outcomes.

### 2.2. Treatment

Patients were treated with chemoradiation therapy (CRT), and the primary tumor intended radiation dose varied from 50 Gy to 58 Gy depending on T stage and nodal size. Patients predominantly received mitomycin (MMC) and fluorouracil (5-FU) infusion or oral capecitabine as concurrent chemotherapy. MMC was administered during weeks 1 and 5 of treatment, infusional 5-FU was administered during weeks 1 and 5, and capecitabine was administered orally twice daily on radiation treatment days. Following CRT, patients were surveilled with anoscopy every 6 to 12 months and annual imaging with CT or MRI for 3 years. Local failure after CRT was defined as persistent disease at 6 months after CRT, local recurrence, and progression of disease during therapy. Patients who had persistent disease within 6 months of finishing chemoradiation were recommended to undergo surgery.

Surgical treatment of local failure included APR, extended APR, or pelvic exenteration. Extended APR involved performing a standard APR procedure with the resection of contents from one additional pelvic compartment or a lateral or inguinal lymph node dissection. Surgeries that included a cystectomy and APR were considered total pelvic exenterations. Indications for an extended APR were tumor extension to the pelvic sidewall or adjacent organs such as the posterior vaginal wall or the prostate. Involvement or close proximity (within 1–2 mm) of the tumor to the urethra or bladder was an indication of pelvic exenteration.

### 2.3. Statistical Analysis

Statistical analysis was performed using SPSS version 27 (IBM). Frequencies and percentages were calculated for categorical variables, and medians and ranges were calculated for continuous variables.

A Kaplan–Meier analysis was performed to estimate 3-year disease-specific survival (DSS), post-APR relapse-free survival (RFS), and disease-free survival (DFS) of the cohort. Multivariate logistic regression analysis was performed to evaluate the effect of several clinical and pathological variables on RFS; a *p*-value < 0.05 was considered statistically significant.

This paper was prepared in accordance with the Strengthening the Reporting of Observational Studies in Epidemiology guidelines [16].

## 3. Results

### 3.1. Patients and Tumor Characteristics

A total of 104 patients with anal SCC underwent APR during the study period, 96 of whom were included in the analysis. Patients with synchronous tumors (*n* = 2), stage IV disease (*n* = 2), missing follow-up information (*n* = 2), or who had upfront surgery (*n* = 2) were excluded. Patient and tumor characteristics are summarized in Table 1.

The median age was 63 years (range 33–87), and 39 (40.6%) patients were male. Seventeen (17.7%) of the patients tested positive for the human immunodeficiency virus (HIV). The indication for salvage APR was persistent disease in 39 (40.6%) patients and local recurrence in 57 (59.4%) patients. All but 10 patients received MMC or cisplatin and 5-FU or capecitabine-based CRT. The remaining patients had either RT alone or 5-FU and CRT (Table 1). The median radiation dose was 54 Gy (range 28–70 Gy), and the median follow-up time was 22 months (IQR 11–47). Seven (7.3%) of the patients had a complete pathologic response after CRT, yet most patients had pathologic stage III disease (*n* = 49; 51.0%) in their surgical specimens. Twenty (20.8%) patients had microscopic tumors or R1 disease, and 19 (19.8%) patients had positive lymph nodes in their surgical specimens. Six (6.3%) patients underwent adjuvant chemotherapy with or without radiation.

Of the patients in our cohort, 20 had targeted tumor-sequencing genetic testing available (MSK-IMPACT), 15 of which were from primary anal SCC, and 5 were from recurrent sites. Nine patients had PIK3CA mutations, seven of whom experienced a recurrence.

### 3.2. Surgical Technique and Complications

The majority of patients underwent an APR alone (*n* = 43, 44.8%) or an extended APR combined with a posterior vaginectomy, prostatectomy, coccygectomy, pelvic sidewall dissection, or inguinal lymph node dissection (*n* = 44; 45.8%). Five (5.2%) patients underwent a posterior pelvic exenteration, and four (4.2%) had a total pelvic exenteration. Two patients (2%) underwent inguinal lymph node resection due to PET-avidity on preoperative imaging, both of which were confirmed to be metastatic lymphadenopathy on final pathology. Six (6.3%) patients had intraoperative radiation therapy. Most patients had a vertical rectus abdominis musculocutaneous (VRAM) flap for perineal reconstruction (Table 2).

The thirty-day morbidity rate was 28.1% (*n* = 27), with 20.8% (*n* = 20) of the cohort experiencing at least a grade 3 complication. The most common adverse event was wound dehiscence, occurring in 10 (10.4%) patients (Table 2). The rate of serious (Clavien–Dindo grade ≥ 3) was unrelated to the extent of surgery; in other words, patients who underwent APR had similar 30-day complication rates to those who had a more extensive operation (OR 0.88, 95% CI 0.321–2.40, *p* = 0.801).

### 3.3. Disease-Specific, Recurrence-Free, and Disease-Free Survival

**Figure 1:**
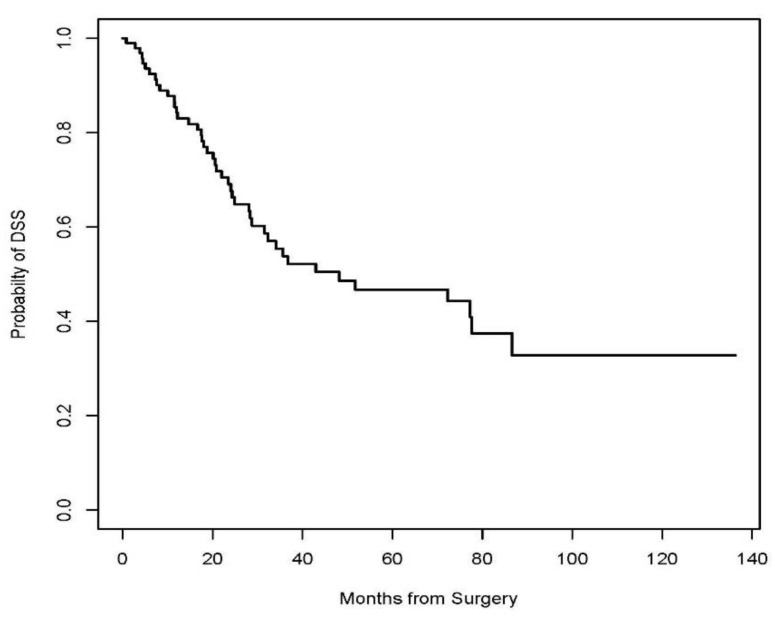
Kaplan–Meier curve for Disease-Specific Survival (DSS).

**Figure 2:**
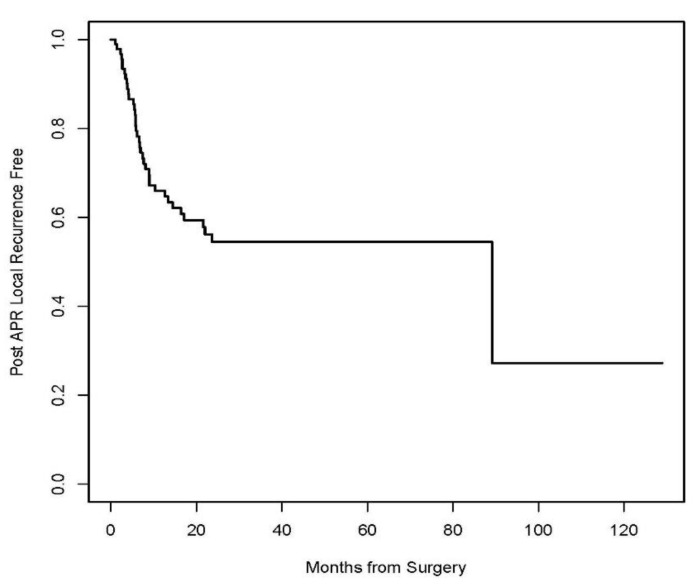
Kaplan–Meier curve for post-APR local recurrence-free survival (RFS).

**Figure 3:**
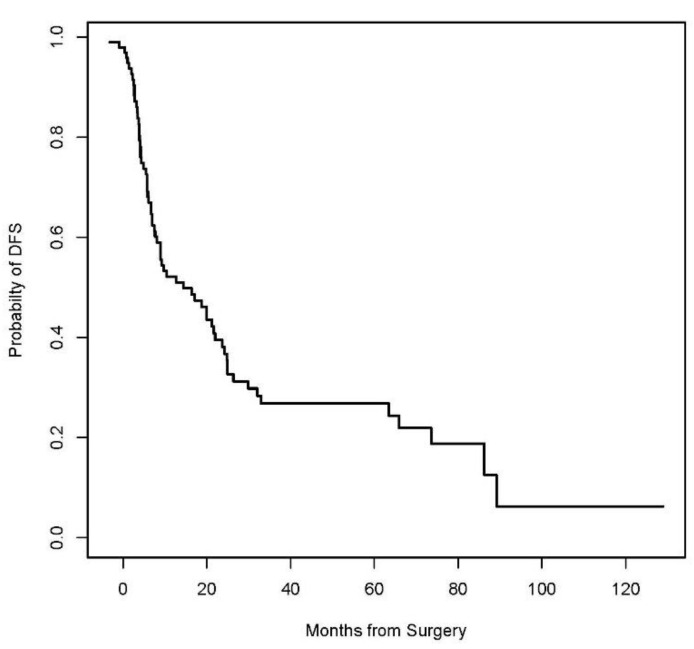
Kaplan–Meier curve for Disease-free survival (DFS).

A Kaplan–Meier Survival analysis was performed to evaluate DSS, RFS, and DFS. With a median follow-up of 22 months (IQR 11 to 47), the 3-year disease-specific survival (DSS), post-APR local recurrence-free survival (RFS), and disease-free survival (DFS) were 53.8% (95% CI 43.5 to 66.5%), 54.5% (95% CI 44.4 to 66.8%), and 26.8% (95% CI 18.6 to 38.7%), respectively (Figure 1, Figure 2 and Figure 3, respectively). Patients who had a complete pathologic response upon surgical resection did not experience a local recurrence or disease-related death. Three (50%) of the six patients who underwent IORT experienced local recurrence. According to multivariate logistic regression analysis, positive microscopic margin (OR 10.0, 95% CI 2.16–46.12, *p* = 0.003), positive lymph nodes (OR 9.19, 95% CI 1.99–42.52, *p* = 0.005), and presence of lymphovascular invasion (OR 2.61, 95% CI 1.05–6.51, *p* = 0.04) in the surgical specimen were associated with recurrence of disease after salvage APR. Gender, indication for APR (recurrent vs. persistent disease), HIV status, and extent of surgery or type of reconstruction did not influence survival outcomes.

## 4. Discussion

This is among the largest single institutional studies to present the morbidity, oncological, and survival outcomes after salvage APR for anal SCC in patients who underwent combined modality treatment at a comprehensive cancer center. Positive microscopic margins, positive nodes, and the presence of lymphovascular invasion in the surgical specimen were associated with the recurrence of disease after salvage APR. Gender, indication for APR (recurrent vs. persistent disease), HIV status, extent of surgery, and type of reconstruction did not influence survival outcomes. The results in this study demonstrate a poor 3-year DFS rate of 26.8%, with a 3-year RFS of 54.5% and a 3-year DSS of 53.8%, which fall within the range of overall survival rates reported in the literature, ranging between 30% and 78% [12,17,18,19,20,21,22,23,24].

Anal squamous cell carcinoma is a rare disease with excellent long-term survival rates of up to 90% in patients who respond to combined modality therapy. Locoregional failure rates, however, are up to 30%, and the effectiveness of salvage APR for anal SCC remains limited [19,22]. Despite refinements in technique and advances in surgical care, oncologic outcomes of salvage APR have remained similar at our center over the past three decades [25,26,27]. Surgical complications in our study were similar to those reported in the literature, with 20% of patients in our cohort experiencing at least a grade 3 complication and wound dehiscence occurring in 53% of patients, despite a high proportion of patients (84%) undergoing flap reconstruction of the perineum [28].

Previous studies have identified heterogeneous risk factors for local and distant recurrence after salvage APR, with some reporting persistent disease as a risk factor while others do not [13,22,29,30]. The largest cohort to address the timing of salvage APR was collected from the National Cancer Database (NCDB) by Fields et al. and included 437 patients treated between 2004 and 2013. The results yielded no significant differences in overall survival between patients who underwent salvage APR within 6 months of CRT (early) and beyond 6 months of treatment (late) [31]. Our present study corroborates this finding as an indication that APR (recurrent vs. persistent disease) did not influence survival outcomes.

While studies are discordant on certain predictors of oncologic outcomes, most recognize positive lymph nodes and positive resection margins as risk factors for recurrence and poor survival. In our cohort, these factors were independently associated with a two to ten-fold risk of local recurrence. Perhaps the most important prognostic indicator in the setting of salvage surgery for locoregional failure, however, is surgery resulting in negative resection margins. Interestingly, the extent of surgery in our study was unrelated to oncologic outcomes and was not correlated to higher morbidity rates when compared to APR alone. Rather, negative resection margins were strongly associated with improved overall and local recurrence-free survival in this study, as addressed in previous reports [22,24,32]. These results suggest that salvage surgery, including extended resection or pelvic exenteration, is justified, especially when negative margins are expected with a more aggressive surgical intervention [19]. Additionally, consideration for preoperative imaging, such as pelvic MRI to assess local tumor extent prior to surgical resection, may guide surgical planning and predict the need for exenteration, which can lead to higher rates of negative resection margins. Furthermore, in cases of advanced disease, consideration for reirradiation can be made; however, further studies are needed to determine the feasibility, efficacy, and optimal regimen for reirradiation.

Another common prognosticator for post-resection recurrence, identified in the present study as well as others, is positive lymph nodes, serving as a major treatment challenge for this rare disease. Previous trials, including RTOG 92-08 and ACCORD3, showed no improvement in local control with radiation dose escalation in combination with MMC or cisplatin-based CRT [33,34,35,36]. Few retrospective series exist examining adjuvant or multimodal therapies for salvaging local failure of anal SCC, especially in node-positive patients. A few small retrospective reviews examined intraoperative radiation with salvage APR for locoregional failure of anal SCC and found little to no oncologic benefit [27,37,38]. More recent work has described the use of immune checkpoint inhibitors and targeted therapy for primary anal SCC; however, no results have been published as supplements to surgery [39]. Although a small sample, almost half of the patients in our study who had genomic data available had a PIK3CA mutation, speaking to the need for further genomic studies that may help identify high-risk patients and target treatment. Activating mutations in PIK3CA have been reported to arise in 20–25% of human anal cancers, suggesting that this pathway may be a relevant target for therapeutic interventions in the future [40,41].

Like comparable studies of its kind, this study is limited by its retrospective nature and sample size. Information regarding genomic testing was limited. While it is one of the larger single institutional studies of its kind, a larger sample size would have provided greater power to this study. Ultimately, locoregional failure of anal SCC remains a treatment challenge, and further, more robust studies are required to identify potentially beneficial treatments in addition to surgical resection.

## 5. Conclusions

Salvage APR for the locoregional failure of anal SCC has poor oncologic outcomes. Positive resection margins, positive lymph nodes, and the presence of lymphovascular invasion in the resected specimen were risk factors for recurrence and decreased survival. Careful preoperative planning, including pelvic MRI to assess tumor extent, extended surgery such as exenteration in order to achieve negative resection margins, and additional salvage therapies, including preoperative reirradiation or targeted therapies, should be explored to improve the oncologic outcomes in the case of recurrent or persistent anal SCC.

## Figures and Tables

**Table 1:** Patient and tumor characteristics.

Characteristics	*N* = 96 (%)
Median age in years (range)	63 (33.87)
No. (%) male	39 (40.6)
No. (%) HIV ^a^ (+)	17 (17.7)
Primary tumor treatment regimen, *n* (%)	
5-FU/capecitabine + MMC ^b^/cisplatin+ RT	86 (89.6)
5-FU + RT	6 (6.2)
RT alone	4 (4.2)
Indication for surgery	
Persistent	39 (40.6)
Recurrent	57 (59.4)
AJCC ^c^ pathological stage, *n* (%)	
0	7 (7.3)
is	1 (1.0)
I	7 (7.7)
II	32 (33.3)
III	49 (51.0)
Pathological T classification, *n* (%)	
pT0/Tis	9 (9.4)
pT1	7 (7.3)
pT2	31 (32.3)
pT3	18 (18.7)
pT4	31 (32.3)
Pathological N classification, *n* (%)	
pN−	77 (80.2)
pN+	19 (19.8)

^a^ HIV, Human Immunodeficiency Virus; ^b^ Mitomycin C; ^c^ AJCC, American Joint Committee on Cancer.

**Table 2:** Surgical Intervention and Complications.

Characteristics	*N* = 96 (%)
No. (%) type surgical approach	
APR	43 (44.8)
Extended APR ^a^	44 (45.8)
Pelvic Exenteration, posterior ^b^	5 (5.2)
Pelvic Exenteration, Total	4 (4.2)
No. (%) type perineal wound closure	
Primary	15 (15.6)
Gluteal Flap	8 (8.3)
Gracilis Flap	4 (4.2)
VRAM	69 (71.9)
No. (%) surgical complications by Clavien–Dindo grade	
1–2	8 (8.3)
3–5	19 (19.8)
No. (%) positive margin resection	20 (20.8)

^a^ Includes rectum and any of the following: partial vaginectomy, prostatectomy, pelvic sidewall dissection, coccygectomy, or inguinal lymph node dissection. ^b^ Includes APR with a total hysterectomy and bilateral salpingo–oophorectomy.

## Data Availability

Data is stored in a secure institutional server. It is not publicly available.

## References

[1] Siegel R.L., Miller K.D., Wagle N.S., Jemal A. (2023). Cancer statistics, 2023. CA Cancer J. Clin..

[2] UKCCCR Anal Cancer Trial Working Party (1996). Epidermoid anal cancer: Results from the UKCCCR randomised trial of radiotherapy alone versus radiotherapy, 5-fluorouracil, and mitomycin. UKCCCR Anal Cancer Trial Working Party. UK Co-ordinating Committee on Cancer Research. Lancet.

[3] Bartelink H., Roelofsen F., Eschwege F., Rougier P., Bosset J.F., Gonzalez D.G., Peiffert D., van Glabbeke M., Pierart M. (1997). Concomitant radiotherapy and chemotherapy is superior to radiotherapy alone in the treatment of locally advanced anal cancer: Results of a phase III randomized trial of the European Organization for Research and Treatment of Cancer Radiotherapy and Gastrointestinal Cooperative Groups. J. Clin. Oncol. Off. J. Am. Soc. Clin. Oncol..

[4] Flam M., John M., Pajak T.F., Petrelli N., Myerson R., Doggett S., Quivey J., Rotman M., Kerman H., Coia L. (1996). Role of mitomycin in combination with fluorouracil and radiotherapy, and of salvage chemoradiation in the definitive nonsurgical treatment of epidermoid carcinoma of the anal canal: Results of a phase III randomized intergroup study. J. Clin. Oncol. Off. J. Am. Soc. Clin. Oncol..

[5] Gunderson L.L., Winter K.A., Ajani J.A., Pedersen J.E., Moughan J., Benson A.B., Thomas C.R., Mayer R.J., Haddock M.G., Rich T.A. (2012). Long-term update of US GI intergroup RTOG 98-11 phase III trial for anal carcinoma: Survival, relapse, and colostomy failure with concurrent chemoradiation involving fluorouracil/mitomycin versus fluorouracil/cisplatin. J. Clin. Oncol. Off. J. Am. Soc. Clin. Oncol..

[6] James R.D., Glynne-Jones R., Meadows H.M., Cunningham D., Myint A.S., Saunders M.P., Maughan T., McDonald A., Essapen S., Leslie M. (2013). Mitomycin or cisplatin chemoradiation with or without maintenance chemotherapy for treatment of squamous-cell carcinoma of the anus (ACT II): A randomised, phase 3, open-label, 2 × 2 factorial trial. Lancet Oncol..

[7] Ko G., Sarkaria A., Merchant S.J., Booth C.M., Patel S.V. (2019). A systematic review of outcomes after salvage abdominoperineal resection for persistent or recurrent anal squamous cell cancer. Color. Dis. Off. J. Assoc. Coloproctol. Great Br. Irel..

[8] Park I.J., Chang G. (2020). Survival and Operative Outcomes After Salvage Surgery for Recurrent or Persistent Anal Cancer. Ann. Coloproctol..

[9] Deshmukh A.A., Suk R., Shiels M.S., Sonawane K., Nyitray A.G., Liu Y., Gaisa M.M., Palefsky J.M., Sigel K. (2020). Recent Trends in Squamous Cell Carcinoma of the Anus Incidence and Mortality in the United States, 2001–2015. J. Natl. Cancer Inst..

[10] Glynne-Jones R., Northover J.M., Cervantes A., ESMO Guidelines Working Group (2010). Anal cancer: ESMO Clinical Practice Guidelines for diagnosis, treatment and follow-up. Ann. Oncol. Off. J. Eur. Soc. Med. Oncol..

[11] van der Wal B.C., Cleffken B.I., Gulec B., Kaufman H.S., Choti M.A. (2001). Results of salvage abdominoperineal resection for recurrent anal carcinoma following combined chemoradiation therapy. J. Gastrointest. Surg. Off. J. Soc. Surg. Aliment. Tract.

[12] Papaconstantinou H.T., Bullard K.M., Rothenberger D.A., Madoff R.D. (2006). Salvage abdominoperineal resection after failed Nigro protocol: Modest success, major morbidity. Color. Dis. Off. J. Assoc. Coloproctol. Great Br. Irel..

[13] Hagemans J.A.W., Blinde S.E., Nuyttens J.J., Morshuis W.G., Mureau M.A.M., Rothbarth J., Verhoef C., Burger J.W.A. (2018). Salvage Abdominoperineal Resection for Squamous Cell Anal Cancer: A 30-Year Single-Institution Experience. Ann. Surg. Oncol..

[14] Patel S.V., Ko G., Raphael M.J., Booth C.M., Brogly S.B., Kalyvas M., Li W., Hanna T. (2020). Salvage Abdominoperineal Resection for Anal Squamous Cell Carcinoma: Use, Risk Factors, and Outcomes in a Canadian Population. Dis. Colon Rectum.

[15] Smith A.J., Whelan P., Cummings B.J., Stern H.S. (2001). Management of persistent or locally recurrent epidermoid cancer of the anal canal with abdominoperineal resection. Acta Oncol..

[16] von Elm E., Altman D.G., Egger M., Pocock S.J., Gøtzsche P.C., Vandenbroucke J.P. (2007). The Strengthening the Reporting of Observational Studies in Epidemiology (STROBE) statement: Guidelines for reporting observational studies. Lancet.

[17] Alamri Y., Buchwald P., Dixon L., Dobbs B., Eglinton T., McCormick J., Wakeman C., Frizelle F.A. (2016). Salvage surgery in patients with recurrent or residual squamous cell carcinoma of the anus. Eur. J. Surg. Oncol. J. Eur. Soc. Surg. Oncol. Br. Assoc. Surg. Oncol..

[18] Allal A.S., Laurencet F.M., Reymond M.A., Kurtz J.M., Marti M.C. (1999). Effectiveness of surgical salvage therapy for patients with locally uncontrolled anal carcinoma after sphincter-conserving treatment. Cancer.

[19] Brown K.G.M., Solomon M.J., Steffens D., Ng K.S., Byrne C.M., Austin K.K.S., Lee P.J. (2023). Pelvic Exenteration for Squamous Cell Carcinoma of the Anus: Oncological, Morbidity, and Quality-of-Life Outcomes. Dis. Colon Rectum.

[20] Ferenschild F.T., Vermaas M., Hofer S.O., Verhoef C., Eggermont A.M., de Wilt J.H. (2005). Salvage abdominoperineal resection and perineal wound healing in local recurrent or persistent anal cancer. World J. Surg..

[21] Guerra G.R., Kong J.C., Bernardi M.P., Ramsay R.G., Phillips W.A., Warrier S.K., Lynch A.C., Ngan S.Y., Heriot A.G. (2018). Salvage Surgery for Locoregional Failure in Anal Squamous Cell Carcinoma. Dis. Colon Rectum.

[22] Lefèvre J.H., Corte H., Tiret E., Boccara D., Chaouat M., Touboul E., Svrcek M., Lefrancois M., Shields C., Parc Y. (2012). Abdominoperineal resection for squamous cell anal carcinoma: Survival and risk factors for recurrence. Ann. Surg. Oncol..

[23] Mullen J.T., Rodriguez-Bigas M.A., Chang G.J., Barcenas C.H., Crane C.H., Skibber J.M., Feig B.W. (2007). Results of surgical salvage after failed chemoradiation therapy for epidermoid carcinoma of the anal canal. Ann. Surg. Oncol..

[24] Schiller D.E., Cummings B.J., Rai S., Le L.W., Last L., Davey P., Easson A., Smith A.J., Swallow C.J. (2007). Outcomes of salvage surgery for squamous cell carcinoma of the anal canal. Ann. Surg. Oncol..

[25] Ellenhorn J.D., Enker W.E., Quan S.H. (1994). Salvage abdominoperineal resection following combined chemotherapy and radiotherapy for epidermoid carcinoma of the anus. Ann. Surg. Oncol..

[26] Beal K.P., Wong D., Guillem J.G., Paty P.B., Saltz L.L., Wagman R., Minsky B.D. (2003). Primary adenocarcinoma of the anus treated with combined modality therapy. Dis. Colon Rectum.

[27] Wright J.L., Gollub M.J., Weiser M.R., Saltz L.B., Wong W.D., Paty P.B., Temple L.K., Guillem J.G., Minsky B.D., Goodman K.A. (2011). Surgery and high-dose-rate intraoperative radiation therapy for recurrent squamous-cell carcinoma of the anal canal. Dis. Colon Rectum.

[28] Chessin D.B., Hartley J., Cohen A.M., Mazumdar M., Cordeiro P., Disa J., Mehrara B., Minsky B.D., Paty P., Weiser M. (2005). Rectus flap reconstruction decreases perineal wound complications after pelvic chemoradiation and surgery: A cohort study. Ann. Surg. Oncol..

[29] Kitaguchi D., Tsukada Y., Ito M., Horasawa S., Bando H., Yoshino T., Yamada K., Ajioka Y., Sugihara K. (2023). Survival outcomes following salvage abdominoperineal resection for recurrent and persistent anal squamous cell carcinoma. Eur. J. Surg. Oncol. J. Eur. Soc. Surg. Oncol. Br. Assoc. Surg. Oncol..

[30] Severino N.P., Chadi S.A., Rosen L., Coiro S., Choman E., Berho M., Wexner S.D. (2016). Survival following salvage abdominoperineal resection for persistent and recurrent squamous cell carcinoma of the anus: Do these disease categories affect survival?. Color. Dis..

[31] Fields A.C., Melnitchouk N., Senturk J., Irani J., Bleday R., Goldberg J. (2019). Early versus late salvage abdominoperineal resection for anal squamous cell carcinoma: Is there a difference in survival?. J. Surg. Oncol..

[32] Correa J.H., Castro L.S., Kesley R., Dias J.A., Jesus J.P., Olivatto L.O., Martins I.O., Lopasso F.P. (2013). Salvage abdominoperineal resection for anal cancer following chemoradiation: A proposed scoring system for predicting postoperative survival. J. Surg. Oncol..

[33] Ajani J.A., Winter K.A., Gunderson L.L., Pedersen J., Benson A.B., Thomas C.R., Mayer R.J., Haddock M.G., Rich T.A., Willett C. (2008). Fluorouracil, mitomycin, and radiotherapy vs fluorouracil, cisplatin, and radiotherapy for carcinoma of the anal canal: A randomized controlled trial. JAMA.

[34] John M., Pajak T., Flam M., Hoffman J., Markoe A., Wolkov H., Paris K. (1996). Dose escalation in chemoradiation for anal cancer: Preliminary results of RTOG 92-08. Cancer J. Sci. Am..

[35] Konski A., Garcia M., John M., Krieg R., Pinover W., Myerson R., Willett C. (2008). Evaluation of planned treatment breaks during radiation therapy for anal cancer: Update of RTOG 92-08. Int. J. Radiat. Oncol. Biol. Phys..

[36] Peiffert D., Tournier-Rangeard L., Gérard J.P., Lemanski C., François E., Giovannini M., Cvitkovic F., Mirabel X., Bouché O., Luporsi E. (2012). Induction chemotherapy and dose intensification of the radiation boost in locally advanced anal canal carcinoma: Final analysis of the randomized UNICANCER ACCORD 03 trial. J. Clin. Oncol. Off. J. Am. Soc. Clin. Oncol..

[37] Hallemeier C.L., You Y.N., Larson D.W., Dozois E.J., Nelson H., Klein K.A., Miller R.C., Haddock M.G. (2014). Multimodality therapy including salvage surgical resection and intraoperative radiotherapy for patients with squamous-cell carcinoma of the anus with residual or recurrent disease after primary chemoradiotherapy. Dis. Colon Rectum.

[38] Osborne M.C., Maykel J., Johnson E.K., Steele S.R. (2014). Anal squamous cell carcinoma: An evolution in disease and management. World J. Gastroenterol..

[39] Carr R.M., Jin Z., Hubbard J. (2021). Research on Anal Squamous Cell Carcinoma: Systemic Therapy Strategies for Anal Cancer. Cancers.

[40] Cacheux W., Rouleau E., Briaux A., Tsantoulis P., Mariani P., Richard-Molard M., Buecher B., Dangles-Marie V., Richon S., Lazartigues J. (2016). Mutational analysis of anal cancers demonstrates frequent PIK3CA mutations associated with poor outcome after salvage abdominoperineal resection. Br. J. Cancer.

[41] Cacheux W., Dangles-Marie V., Rouleau E., Lazartigues J., Girard E., Briaux A., Mariani P., Richon S., Vacher S., Buecher B. (2018). Exome sequencing reveals aberrant signalling pathways as hallmark of treatment-naive anal squamous cell carcinoma. Oncotarget.

